# Determination of Dabigatran Concentration in Human Plasma and Breast Milk

**DOI:** 10.1155/2021/5949385

**Published:** 2021-10-22

**Authors:** F. Sidgwick, A. Porter, P. Ayuk, F. Kamali, A. Truemann

**Affiliations:** ^1^Newcastle University Protein and Proteome Analysis (NUPPA), Newcastle Upon Tyne, UK; ^2^Newcastle Upon Tyne Hospitals Foundation Trust, Newcastle Upon Tyne, UK; ^3^Translational and Clinical Research Institute, Newcastle University and Newcastle Upon Tyne Hospitals Foundation Trust, Newcastle Upon Tyne, UK

## Abstract

Venous thromboembolism (VTE) is an important cause of death following childbirth. Dabigatran etexilate can be a useful prophylaxis in susceptible women during the postpartum period. However, it is not clear whether dabigatran is excreted into breast milk in amounts which can be harmful to the suckling baby. We have developed an accurate, sensitive, and specific assay for the quantitation of dabigatran in both human plasma and breast milk. This is particularly useful for the determination of the extent by which dabigatran is secreted into breast milk in relation to its systemic availability. Dabigatran was enriched from both matrices using solid-phase extraction prior to separation on a C8-RPLC column and detection using SRM on a QqTrap mass spectrometer. The assay was validated for specificity, sensitivity, linearity, precision, accuracy, and stability of the analyte in human plasma and breast milk. The lower limit of detection for dabigatran was 20 pg/ml in plasma and 75 pg/ml in breast milk. This assay will aid future studies for the measurement of dabigatran concentrations in human breast milk to help determine if dabigatran etexilate can safely be administered to breast-feeding women.

## 1. Introduction

Venous thromboembolism (VTE) is an important cause of death following childbirth [1]. Dabigatran etexilate, a direct thrombin inhibitor, is a member of the non-vitamin K antagonist oral anticoagulants (NOACs) used for the treatment and prophylaxis of thromboembolic disorders [2–4]. Dabigatran etexilate is rapidly absorbed following oral ingestion with mean peak plasma concentrations (*C*_max_ = 160 ng/ml) of the active agent, dabigatran, reaching within 2 h of administration [5]. Dabigatran etexilate is currently not recommended for use as a prophylaxis in women susceptible to thromboembolism during the postpartum period. This is because it is not known at this stage whether dabigatran is excreted into breast milk in amounts which can be harmful to the suckling baby. Investigations exploring dabigatran secretion into breast milk will require the use of a sensitive and robust assay. To date, several analytical methods for determining dabigatran concentrations in human plasma samples have been reported, most of which utilise liquid chromatography-mass spectrometry (LC-MS) because of its superior accuracy, sensitivity, and specificity [6–12]. These assays have a LC turnaround time of 4.5–7 minutes and a lower limit of quantification (LOQ) between 25 pg/ml and 0.3 ng/ml [6–12]. However, there is no assay reported which measures dabigatran concentration in human breast milk. Here, we report a flexible, robust, rapid, and sensitive assay that can be utilised to determine dabigatran concentration in both plasma and breast milk over a concentration range of three orders of magnitude. This assay is particularly useful for future studies aimed at determining the extent by which dabigatran is secreted into breast milk in relation to its systemic availability in postpartum women.

## 2. Materials and Methods

Expressed human breast milk that was surplus to requirement was donated for use in this study. Dabigatran and ^13^C_6_-dabigatran were obtained from Alsachim (France). Agilent Bond Elut C18 SPE cartridges (1 mL) were obtained from Crawford Scientific (UK). Formic acid and HPLC-grade acetonitrile were obtained from Fisher Scientific (UK). An Ace 5 C8-300 column (250 × 1 mm) was supplied by Advanced Chromatography Technologies, UK. All other solvents used were of analytical grade and were obtained from VWR (UK). All the stock standard solutions, calibration standards, and quality control samples of dabigatran were prepared using a calibrated accurate weighing balance. Dilutions were prepared from stock solutions on the day of analysis. The dabigatran concentration range chosen for the standard curve allowed for the variance in plasma dabigatran concentrations observed in both healthy volunteers [5] and patient populations [13] following the administration of a therapeutic dose of dabigatran etexilate.

^13^C_6_-dabigatran (2.5 ng in 5 *μ*l of water) was added to 500 *μ*l of breast milk or plasma. Samples were centrifuged in an Eppendorf benchtop centrifuge at 13,000 rpm for 5 min at room temperature. 200 *μ*l of the aqueous layer or 200 *μ*l of plasma were applied to a Bond Elute C18 1 ml SPE cartridge that had been conditioned sequentially with 500 *μ*l of methanol and 500 *μ*l of HPLC grade water. Cartridges were washed with 500 *μ*l of HPLC grade water and eluted into glass tubes first with 500 *μ*l of methanol and then with 500 *μ*l of propan-2-ol. The eluent was dried in a nitrogen stream at room temperature prior to redissolving in 200 *μ*l of 0.1% formic acid in HPLC-grade water. The resulting solution was centrifuged at 13,000 rpm for 2 min at room temperature in an Eppendorf bench centrifuge prior to transfer into a glass HPLC vial.

### 2.1. LCMSMS Analysis

The resultant solution (1 *μ*l) from each vial was injected onto a Dionex Ultimate 3000 HPLC system (Thermo Fisher, UK), equipped with an Ace 5 C8-300 column (250 mm × 1 mm, Advanced Chromatography Technologies, UK) equilibrated with 4% B (HPLC solvents: A: 0.1% formic acid in HPLC-grade water; B: acetonitrile, and C: 0.1% formic acid in methanol) at a flow rate of 50 *μ*l/min through the loading pump of the HPLC system. The column was eluted using the gradient shown in Table 1, and the eluent was monitored online using a QTrap 4000 mass spectrometer (Sciex, UK).

Source parameters of the QTrap mass spectrometer were as follows: entrance potential 12, curtain gas 25, CAD 4, ion source voltage 5 kV, temperature 200°C, GS1 20, GS2 20, and interface heater ON. Six SRM transitions were monitored as described in Section 3. N2 was used as the curtain gas, nebuliser, and collision gas.

## 3. Results

### 3.1. LCMSMS Optimisation

**Figure 1:**
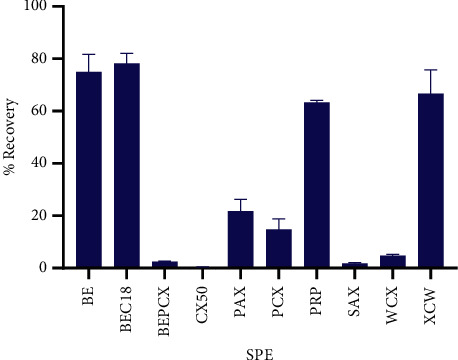
Product ion spectra of dabigatran (a) and ^13^C_6_-dabigatran (b). ^13^C_6_-carbon atoms in the internal standard are indicated as red dots in the structures.

**Figure 2:**
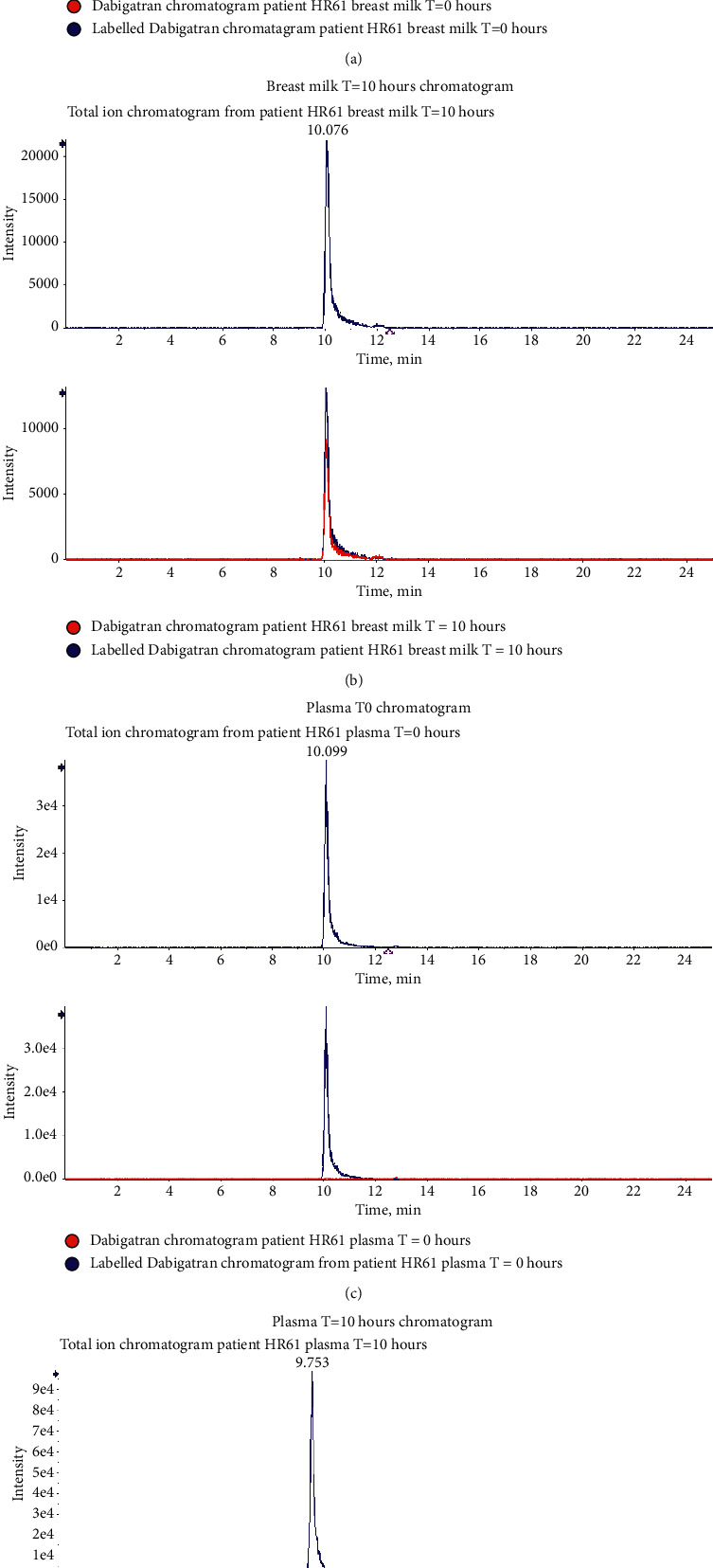
Chromatograms of dabigatran extracted from plasma and breast milk obtained from a patient before (*T* = 0) and 10 h (*T* = 10 h) after the ingestion of dabigatran etexilate. (a) Breast milk *T*0 chromatogram, (b) breast milk *T* = 10 hours chromatogram, (c) plasma *T*0 chromatogram, and (d) plasma *T* = 10 hours chromatogram.

Dabigatran and the ^13^C-labelled dabigatran standard were infused into a QTrap4000 mass spectrometer using positive mode electrospray ionisation. After optimisation of source parameters, product ion spectra were recorded (Figure 1) and three transitions were chosen for both the internal standard and the unlabelled analyte (Tables 2 and 3), and collision energy and source parameters were optimised separately for each transition. Following MS optimisation, samples were separated using reversed-phase HPLC (RP-HPLC) on an Ace C8 column and a 15 min gradient from 4% acetonitrile to 95% acetonitrile.  Figure 2 shows the chromatogram in plasma and breast milk obtained from a postpartum woman following the oral administration of 220 mg of dabigatran etexilate.

### 3.2. Solid-Phase Extraction

To extract dabigatran from either milk or plasma, ten different solid-phase extraction columns were evaluated for recovery and reproducibility (supplementary figure S1). The best performance (76% recovery of dabigatran) was obtained using Agilent 30 mg 1 ml BondElute C18 SPE cartridges.

### 3.3. Specificity and Sensitivity

**Figure 3:**
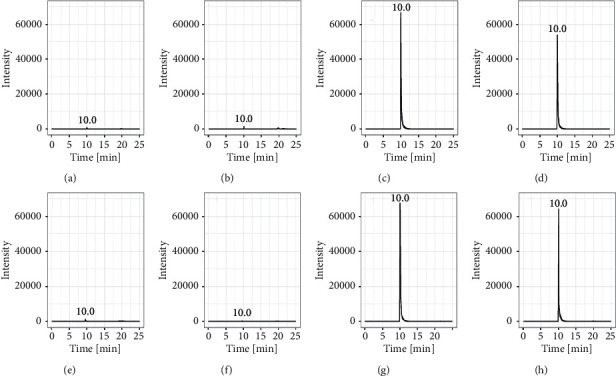
Extracted ion chromatograms of blank plasma (a), blank breast milk (b), plasma spiked with ^13^C_6_-dabigatran (c), breast milk spiked with ^13^C_6_-dabigatran (d), plasma spiked with ^13^C_6_-dabigatran and dabigatran (e), and breast milk spiked with ^13^C_6_-dabigatran and dabigatran (f).

Specificity and sensitivity were tested using chromatograms of blank breast milk and blank plasma samples and samples of both matrices spiked with 0.01 ng dabigatran. Neither matrix showed interference at the retention time of dabigatran (Figure 3).

### 3.4. Linearity and Dynamic Range

**Figure 4:**
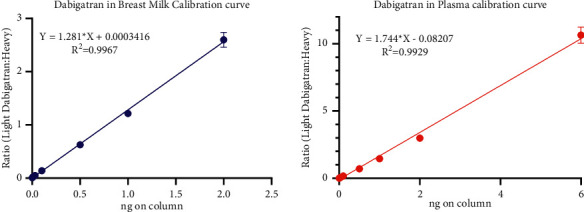
Calibration curves for dabigatran in plasma (concentration range: 0.0005 ng–6 ng) (a) and breast milk (concentration range: 0.0005–2 ng) (b).

The assay was linear (*R*^2^ > 0.99, *n* = 9 data points) for dabigatran in breast milk and plasma over a range of 5 ng/ml to 2 mg/ml.  Figure 4 shows typical standard curves for dabigatran constructed in plasma and breast milk. During the acquisition of the measured values for dabigatran in breast milk, a blank was run as every third sample while data for a standard curve and data for the breast milk samples were acquired. The mean ± SE dabigatran area in all blanks (*n* = 24) was 3210 ± 435, and the mean ± SE ^13^C-labelled dabigatran area in all breast milk standard samples (0.5 nmol of dabigatran on column; *n* = 33) was 720773 ± 27011. This implies that the noise level (including potential carryover) is 2.2 pmol on column, corresponding to a breast milk dabigatran concentration of 20 pg/ml. Defining the lower limit of detection (LLOD) as three times the noise level yielded an LLOD of 75 pg/ml. Defining the lower limit of quantitation (LLOQ) as ten times the noise level yielded an LLOD of 2200 pg/ml.

### 3.5. Precision and Accuracy

Using the optimised assay, plasma and breast milk samples were spiked with 0.5 ng of dabigatran and analysed in triplicate on the same day and on separate days in order to determine intraday and interday reproducibility (Table 4).

### 3.6. Stability of Dabigatran Samples

To determine if dabigatran is undergoing biological or chemical modification during storage in breast milk, triplicate aliquots of dabigatran were subjected to a variety of different treatments, including a single and a double freeze-thaw cycle (−80°C), storage for 20 h or 44 h at 4°C, and storage at room temperature for 1 h, 4 h, or 20 h. The results as shown in Table 5 indicate that none of these treatments was associated with a change in dabigatran concentration.

## 4. Discussion

We have developed a highly sensitive and reproducible assay to determine dabigatran concentration in human plasma and breast milk. The assay is linear over more than three orders of magnitude. It takes four hours to prepare a batch of 24 samples plus 30 min per sample for the LCMSMS analysis on a Sciex Qtrap 4000 mass spectrometer, an instrument that is frequently available in many clinical laboratories. To our knowledge, this is the first published procedure to determine dabigatran concentration in human breast milk samples.

Dabigatran etexilate is currently licensed for the prevention of venous thromboembolism (VTE) following hip and knee surgery. VTE is an important cause of death following childbirth [1], and current preventive strategies include the administration of injectable heparin [1]. Dabigatran etexilate is administered orally and would, therefore, have advantages over injectable heparin. This assay will aid future studies to determine if dabigatran is detectable in breast milk in clinically significant concentrations following oral administration of dabigatran etexilate to breast-feeding women. Such studies are essential if dabigatran etexilate is to be considered as an alternative to injectable heparin. Our assay permits the detection of very low concentrations of dabigatran in breast milk and ensures that clinical implications can be fully evaluated before dabigatran etexilate is considered for use in breast-feeding women.

## Figures and Tables

**Table 1:** Gradient programme on the Dionex Ultimate HPLC system using an Ace 5 C8-300 column (250 mm × 1 mm).

Minutes	Flow (*μ*L/min)	% B	% C
0.0	50	4	0
1.5	50	4	0
2.5	50	15	0
10.0	50	40	0
12.5	50	65	0
14.0	50	95	0
14.1	50	95	0
14.2	50	4	0
15.0	50	4	96
15.8	50	4	96
17.0	50	4	0
25.0	50	4	0

**Table 2:** MRM transitions monitored for dabigatran.

Q1 mass	Q3 mass	Time (msec)	DP	CE
472.2	289.2	50	96	41
472.2	306.3	50	96	29
472.2	324.0	50	101	29

**Table 3:** MRM transitions monitored for ^13^C dabigatran.

Q1 mass	Q3 mass	Time (msec)	DP	CE
478.2	295.2	50	96	41
478.2	312.4	50	96	29
478.2	330.3	50	101	29

**Table 4:** Intraday and interday reproducibility.

Dabigatran concentration (ng/ml)	Intraday CV (%)	Interday CV (%)
*Milk*		
10	6.22	18.68
30	8.40	18.98
100	4.37	6.97
500	2.42	9.05
1000	2.67	2.41
2000	0.39	3.43

*Plasma*		
3	19.43	38.75
10	13.89	35.76
30	4.83	7.00
100	2.84	7.27
500	2.76	2.86
1000	3.06	4.21
2000	2.84	6.35

**Table 5:** Stability testing of triplicates of a 300 *μ*l aliquot of breast milk containing 48 pg of dabigatran. Three technical replicates were treated as indicated, and dabigatran concentration was determined. ANOVA indicated that none of the treatments showed a significant difference in dabigatran concentration. Frozen samples were stored at −20°C for one week. RT = room temperature.

Treatment	Dabigatran (pg)	*n*
None	52.7	3
Freeze, thaw, freeze	44.5	2
4°C, 44 h	46.0	3
4°C, 20 h	47.0	3
RT, 20 h	47.3	3
RT, 4 h	42.0	3
RT, 1 h	41.3	3
−20°C, 1 week	46.7	3

## Data Availability

Experimental data will be made available on request.

## Supplementary Materials

Supplementary Materials Supplementary figure S1: graph to show the 10 different SPE cartridges trialled, tested in triplicate for reproducibility and recovery.
