# Supplementary figures and images for: Determination of Dabigatran Concentration in Human Plasma and Breast Milk
Source: Int J Anal Chem. 2021 Oct 22;2021:5949385. doi: 10.1155/2021/5949385 (PMC8556115; doi:10.1155/2021/5949385)

## Slide 1
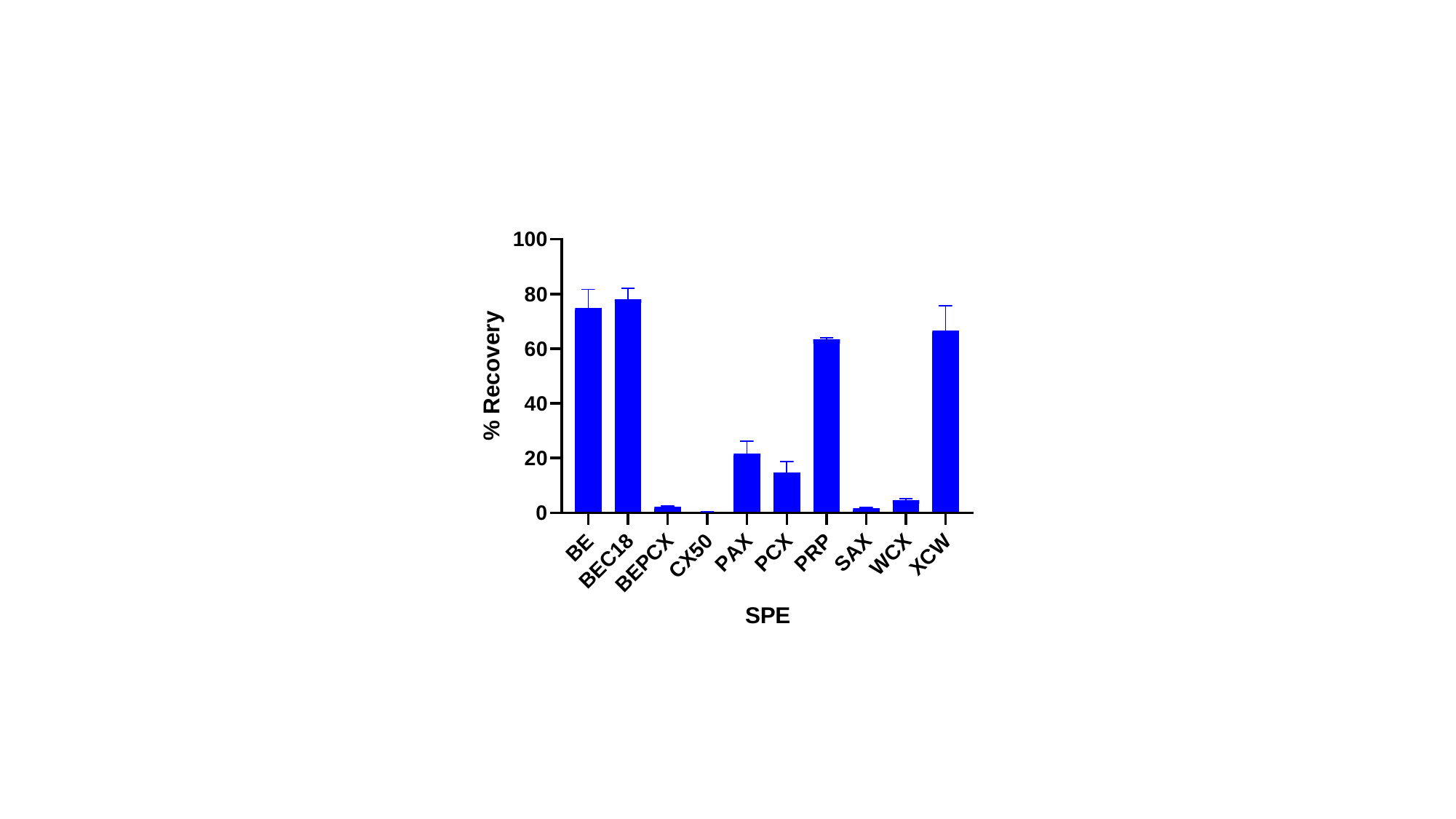

Supplement: Supplementary Materials — Supplementary figure S1: graph to show the 10 different SPE cartridges trialled, tested in triplicate for reproducibility and recovery. [file 5949385.f1.pptx]
